# Short‐ and Long‐Term Swallowing Outcomes in Head and Neck Cancer Patients Receiving TORS and Adjuvant Therapy

**DOI:** 10.1002/hed.28033

**Published:** 2024-12-23

**Authors:** Abigail C. Weiland, Sandeep Samant, Alex E. Clain, Bonnie Martin‐Harris

**Affiliations:** ^1^ Department of Medicine McGaw Medical Center of Northwestern University Chicago Illinois USA; ^2^ Department of Otolaryngology‐Head and Neck Surgery Northwestern University Feinberg School of Medicine Chicago Illinois USA; ^3^ Roxelyn and Richard Pepper Department of Communication Sciences and Disorders School of Communication Evanston Illinois USA; ^4^ Otolaryngology‐Head and Neck Surgery and Radiation Oncology Feinberg School of Medicine Chicago Illinois USA

**Keywords:** dysphagia, swallowing, TORS, videofluoroscopy

## Abstract

**Background:**

Dysphagia (difficulty swallowing) is a common morbidity resulting from the treatment of head‐and‐neck squamous‐cell carcinoma (HNSCC) due to surgery and chemoradiation. Transoral robotic surgery (TORS) is a minimally invasive surgical technique for the management of HNSCC, which ideally avoids many of the known complications of open surgery. Research describing physiologic swallowing impairment after surgery using videofluoroscopy is lacking.

**Methods:**

We evaluated videofluoroscopic swallowing studies of 37 patients who received TORS for head and neck cancer using a validated scoring tool, the Modified Barium Swallow Impairment Profile (MBSImP), at three time points including baseline.

**Results:**

Patients had worsened physiologic impairments in the immediate post‐operative and late post‐operative periods, particularly in components related to airway protection. Many patients also had baseline swallowing impairment.

**Conclusions:**

Further research is required to elucidate dysphagia at discrete time points before and after treatment as well as with different and evolving adjuvant therapy protocols.

## 1 Introduction

Dysphagia (swallowing dysfunction) is one of the most common negative side effects of the treatment of head‐and‐neck squamous‐cell carcinoma (HNSCC), with studies reporting 40%–80% prevalence [1, 2, 3, 4]. This high prevalence is of critical concern because, in HNSCC populations, the presence and severity of dysphagia is associated with worsened quality of life, anxiety, and depression [5], and is even a cause of mortality among cancer patients due to aspiration pneumonia [6, 7].

Several treatment modalities have been proposed to reduce or prevent dysphagia among these patients compared to traditional therapy, including induction chemotherapy [8], dysphagia‐optimized intensity‐modulated radiotherapy [9], and organ‐sparing surgical treatment [10]. Prophylactic swallowing exercises before treatment have also been proposed as a means to prevent dysphagia [1]. One main organ‐sparing surgical treatment is transoral robotic surgery (TORS), a surgical technique that allows surgeons to remove oropharyngeal tumors in a minimally invasive fashion [11]. One of the primary aims of TORS is to improve speech and swallowing outcomes compared to previously performed techniques [12, 13, 14]. Even so, dysphagia remains one of the primary morbidities in patients receiving TORS, especially in those who also receive adjuvant radiotherapy or chemoradiotherapy [15, 16]. An understanding of dysphagia after TORS is therefore critical to optimize targeted swallow therapy and outcomes for these patients.

A number of studies have evaluated patient‐reported functional outcomes after TORS, but there is limited literature evaluating dysphagia using physiologic metrics based on videofluoroscopy [17, 18]. Outcome measures used in prior studies are primarily subjective and patient‐reported, including the MD Anderson Dysphagia Inventory (MDADI) [19, 20, 21, 22], Functional Oral Intake Scale [23], Eating Assessment Tool (EAT‐10) [24], Performance Status Scale—Head and Neck (PSSHN) [23, 25, 26], and Functional Oral Swallowing Scale [27]. Patient perceptions of impairment can be markedly different from objective physiologic findings [28, 29, 30]. For example, some patients aspirate after TORS without subjective awareness [31]. As such, a physiologic assessment of swallowing function after TORS is critical in understanding true impairment after surgery, which may be missed with patient‐reported dysphagia alone. Furthermore, dysphagia is a symptom, not a diagnosis, which can be caused by many different anatomic problems, so understanding the physiologic cause of impairment is crucial in guiding targeted therapy.

The gold standard objective measure of dysphagia is the Modified Barium Swallow (MBS), a videofluoroscopic evaluation of the anatomy and physiology of the oropharyngeal swallow performed by clinicians [32]. One advantage of MBS evaluation over other measures of dysphagia is that it can be used to characterize drivers of impairment and identify targeted interventions [32, 33, 34, 35]. Numerous metrics have been developed to quantify the type and severity of dysphagia based on MBS [36, 37, 38]; one, the Modified Barium Swallow Impairment Profile (MBSImP), is a validated, standardized assessment tool that quantifies swallowing function with 17 discrete components [39]. Few studies have used MBSImP to characterize dysphagia after TORS compared to baseline [40, 41]. In one study, the authors found the majority of TORS patients to have impairments in six pharyngeal components of MBSImP in the early post‐operative period [40]. Another published study compared physiologic swallowing impairment in TORS patients to those receiving primary radiation therapy 3–6 months post‐treatment using the MBSImP [41]. Although they found similar rates of swallowing impairment in the two groups at 3–6 months, TORS patients were noted to have worse pharyngeal contraction, and primary RT patients were found to have worse laryngeal vestibular closure [41].

The widespread use of adjuvant therapy post‐TORS adds complexity to understanding dysphagia after treatment; at present, over 80% of patients receiving TORS also receive adjuvant therapy [42]. Muscular injury and tissue insult secondary to radiotherapy and chemoradiotherapy can contribute to oropharyngeal dysphagia [3, 43, 44, 45]. TORS is often offered to patients because it is believed to have superior functional outcomes to other treatment modalities, and survival is similar between them [46, 47].

The primary goal of the current study was to characterize swallow impairment physiologically in TORS patients at three time points using the MBSImP: (1) pre‐surgery, (2) post‐surgery and pre‐adjuvant therapy (perioperative period), and (3) post‐adjuvant therapy (post‐acute recovery period). An understanding of the physiologic etiologies of impairment in these patients will be important for providers to anticipate dysphagia morbidity and work with a multidisciplinary team to craft targeted treatment recommendations.

## 2 Materials and Methods

### 2.1 Study Design

This was a single‐center, retrospective cohort study.

### 2.2 Participants

Thirty‐seven subjects were selected for this study who received TORS for head and neck cancer and received videofluoroscopic swallowing evaluations at Northwestern Memorial Hospital between June 2017 and March 2020 (see Table 1 for demographic data).

**TABLE 1:** Demographic data.

Variable	Level	Number (*N* = 37)	(%)
Sex	Female	4	10.8
	Male	33	89.2
Race	Black	1	2.7
	Hispanic	2	5.4
	White	34	91.9
Primary	Tongue base	16	43.2
	Tonsil	21	56.8
T Stage	T1	16	43.2
	T2	16	43.2
	T3	5	13.5
N Stage	N0	3	8.1
	N1	24	64.9
	N2	9	24.3
	N3b	1	2.7
Stage	I	26	70.3
	II	8	21.6
	III	2	5.4
	IVb	1	2.7
Chemoradiation	Radiation only	17	45.9
	Chemoradiation	20	54.1
Age at surgery (years)	Mean	59	
	Median	59	
	Minimum	44	
	Maximum	79	
	SD	8	
# MBS at each time point	Pre‐surgery	29	78.7
	Post‐surgery	29	78.7
	Post‐acute recovery	23	62.2
	All 3	10	27.0

### 2.3 Swallow Studies

Modified Barium Swallow (MBS) studies were completed following the MBSImP protocol for 37 patients. Twenty‐nine subjects had a baseline study before surgery (pre‐operative), averaging 9.5 days prior to surgery. Twenty‐eight subjects completed a short‐term follow‐up videofluoroscopic study after surgery but before completion of chemoradiation/radiation therapy (perioperative), averaging 27.0 days after surgery. Twenty‐six subjects completed a long‐term follow‐up videofluoroscopic swallow study after completion of surgery and chemoradiation/radiation therapy (post‐acute recovery), averaging 218.6 days post‐operatively.

### 2.4 Swallow Outcomes

Archived videofluoroscopic studies were scored by a single rater with approximately 1 year of experience (AW). The rater was not a speech‐language pathologist but was MBSImP‐certified with at least 80% reliability in each component and an average of 87% reliability in all components. The rater was not blinded to the time point. The MBSImP is a validated, reliable scoring tool that rates 17 components of swallow in the categories of oral, pharyngeal, and esophageal swallows [39]. Each component can be scored from 0 to up to 4, with higher scores indicating more impairment (see Table 2 for further details). Twenty percent of the swallow studies were then re‐scored by the same rater to determine intra‐rater reliability. Three subgroups of patients were then stratified based on time points of available swallow studies: (1) pre‐surgery to perioperative period, (2) perioperative period to post‐acute recovery, and (3) pre‐surgery to post‐acute recovery (with or without adjuvant therapy). Change in MBSImP score over the designated time frame was then determined: better (drop in MBSImP score), same, or worse (increase in the MBSImP score).

**TABLE 2:** MBSImP components.

	Number	Physiologic component	Scale
Oral impairment	1	Lip closure	(0–4)
	2	Tongue control/bolus hold	(0–3)
	3	Bolus preparation/mastication	(0–3)
	4	Bolus transport/lingual motion	(0–4)
	5	Oral residue	(0–4)
	6	Initiation of pharyngeal swallow	(0–4)
Pharyngeal impairment	7	Soft palate elevation	(0–4)
	8	Laryngeal elevation	(0–3)
	9	Anterior hyoid excursion	(0–2)
	10	Epiglottic movement	(0–2)
	11	Laryngeal vestibular closure	(0–2)
	12	Pharyngeal stripping wave	(0–2)
	13	Pharyngeal contraction	(0–3)
	14	PES opening	(0–3)
	15	Tongue base retraction	(0–4)
	16	Pharyngeal residue	(0–4)
Esophageal impairment	17	Esophageal clearance	(0–4)

The Penetration Aspiration Scale (PAS) is another validated, reliable tool to describe penetration and aspiration events in videofluoroscopic studies, as well as the patient's response [38]. Like the MBSImP, higher scores indicate more significant impairment: 1 indicates no penetration or aspiration, 2–5 indicate some laryngeal penetration, and 6–8 indicate true aspiration of contrast material into the airway. PAS scores were also given retrospectively by the aforementioned rater and 20% were re‐scored for intra‐rater reliability. Change in the PAS score over time was also determined: better (drop in PAS score), same, and worse (increase in PAS score), over the designated time frames.

### 2.5 Statistical Analysis

For each component, we designated scores as remaining the same, better, or worse over time. We then submitted the “better” and “worse” scores to a binomial test, with the null being that the probability of getting better or worse is equally likely. The *p*‐value was set at 0.05 for significance.

Intra‐rater reliability across all MBSImP components and PAS was calculated using the intra‐class correlation coefficient (ICC) due to each component having differing numbers of severity levels [48]. The resulting intra‐rater reliability was good with ICC = 0.92 (95% CI: 0.90–0.93).

## 3 Results

### 3.1 Pre‐Surgery to Post‐Surgery (Perioperative Period)

**FIGURE 1:**
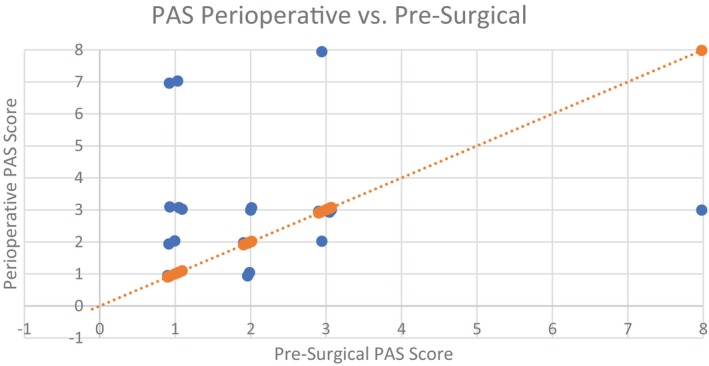
17% improved, 35% remained the same, and 48% worsened. Of those who worsened 64% (7/11) progressed from no penetration/aspiration to penetration OR penetration to aspiration and 18% (2/11) progressed from no penetration/aspiration to aspiration. [Color figure can be viewed at wileyonlinelibrary.com]

Twenty‐three subjects had swallow studies at the first two time points: pre‐surgery and post‐surgery/perioperative period (see Table 3 and Figure 1). Pre‐surgery studies were completed an average of 9.5 days (SD = 8.7 days) before surgery. Post‐surgery swallow studies were completed an average of 27.0 days (SD = 18.1 days) after surgery. Using a binomial test, scores for component 10 (epiglottic movement) and component 11 (laryngeal vestibular closure) were found to significantly worsen from baseline to shortly after surgery (*p* = 0.02 and 0.04, respectively). Component 10 worsened an average of 0.43 points and component 11 worsened an average of 0.39 points.

**TABLE 3:** MBSImP components pre‐surgery vs. post‐surgery.

	c1	c2	c3	c4	c5	c6	c7	c8	c9	c10	c11	c12	c13	c14	c15	c16	c17	PAS
Better	3	2	3	3	2	6	4	2	0	0	2	3	2	5	2	2	6	4
Same	16	16	13	15	15	12	9	13	23	16	11	12	20	14	14	12	6	8
Worse	4	5	4	5	6	6	10	8	0	7	10	8	1	4	7	9	9	11
*p*	1	0.45	1	0.73	0.29	1	0.18	0.11	1	0.02	0.04	0.23	1	1	0.18	0.07	0.61	0.12

### 3.2 Post‐Surgery (Perioperative Period) to Post‐Acute Recovery

**FIGURE 2:**
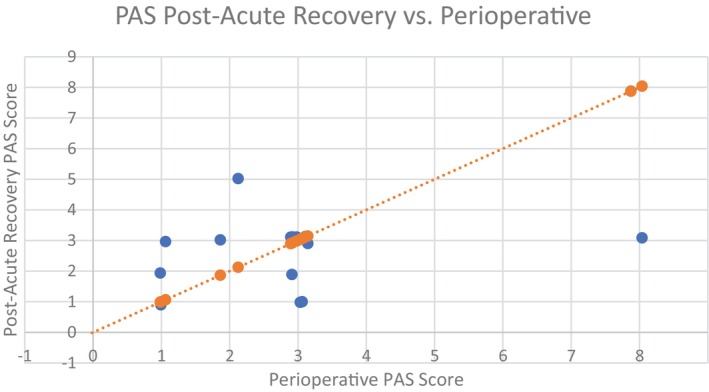
27% improved, 47% remained the same, and 27% worsened. Of those who worsened, 75% (3/4) progressed from no penetration/aspiration to penetration OR penetration to aspiration. [Color figure can be viewed at wileyonlinelibrary.com]

Fifteen subjects had a swallow study both post‐surgically and after adjuvant therapy (post‐acute recovery) (see Table 4 and Figure 2). Post‐surgery swallow studies were completed an average of 27.0 days (SD = 18.1 days) after surgery. Post‐adjuvant therapy studies were completed an average of 218.6 days (SD = 77.8 days) after surgery and 135.4 days (SD = 71.9 days, range = 24–326 days) after completion of adjuvant therapy (radiation therapy or chemoradiation). No components were found to significantly change during this period.

**TABLE 4:** MBSImP components post‐surgery to post‐adjuvant therapy.

	c1	c2	c3	c4	c5	c6	c7	c8	c9	c10	c11	c12	c13	c14	c15	c16	c17	PAS
Better	3	3	0	1	1	3	4	3	1	3	7	3	0	2	2	1	3	4
Same	9	9	13	9	9	8	11	11	14	12	5	7	14	9	11	14	5	7
Worse	3	3	1	5	5	4	0	1	0	0	3	5	0	4	2	0	7	4
*p*	1	1	1	0.83	0.83	1	0.13	0.63	1	0.25	0.34	0.73	1	0.69	1	1	0.34	1

### 3.3 Pre‐Surgery to Post‐Acute Recovery

**FIGURE 3:**
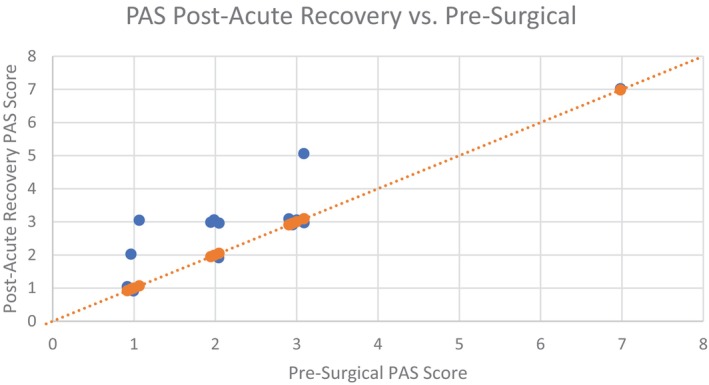
0% improved, 63% remained the same, and 38% worsened. Of those patients who worsened, 50% (3/6) progressed from no penetration/aspiration to penetration OR penetration to aspiration. [Color figure can be viewed at wileyonlinelibrary.com]

Sixteen subjects had a swallow study both pre‐surgically and after adjuvant therapy (see Table 5 and Figure 3). Pre‐surgery studies were completed an average of 9.5 days (SD = 8.7 days) before surgery. Post‐adjuvant therapy studies were completed an average of 218.6 days (SD = 77.8 days) after surgery and 135.4 days (SD = 71.9 days) after completion of adjuvant therapy (radiation therapy or chemoradiation). Component 11 (laryngeal vestibular closure) and the PAS score were found to significantly worsen over this period (*p*‐score = 0.02 and 0.03, respectively). Component 11 worsened an average of 0.44 points during this period, and PAS worsened an average of 0.5 points. Of the 6 patients who experienced a worsening of PAS during this time, two progressed from no penetration/aspiration (PAS score 1) to evidence of penetration (PAS score 2–5) and one progressed from penetration (PAS score 2–5) to aspiration (PAS score 6–8) (see Table 6). The remaining three patients experienced penetration at both time points.

**TABLE 5:** MBSImP components pre‐surgery to post‐acute recovery.

	C1	c2	c3	c4	c5	c6	c7	c8	c9	c10	c11	c12	c13	c14	c15	c16	c17	PAS
Better	3	4	1	1	2	2	0	0	0	2	0	3	2	2	0	1	3	0
Same	9	9	10	8	11	10	12	13	15	13	9	9	13	13	12	10	4	10
Worse	4	3	5	7	3	4	4	3	1	1	7	4	0	1	4	5	8	6
*p*	1	1	0.22	0.07	1	0.69	0.13	0.25	1	1	0.02	1	0.5	1	0.13	0.22	0.23	0.03

**TABLE 6:** Number of patients with PAS scores falling into each of the three categories: No penetration/aspiration (PAS score 1), penetration only (PAS score 2–5), and aspiration (PAS score 6–8) at each of the three time points. There was a slight increase in the percentage of patients experiencing penetration or aspiration after surgery that improved (but not fully) in the post‐acute recovery period.

PAS bin	PAS pre‐surgery	PAS post‐surgery	PAS post‐acute recovery
No penetration/aspiration	9	3	3
Penetration	18	21	17
Aspiration	2	5	3

## 4 Discussion

Dysphagia is one of the primary morbidities affecting the quality of life of head and neck cancer patients during and after treatment, and it is the complication of treatment that is most strongly correlated with the overall quality of life [49, 50]. The prevalence of oropharyngeal cancer continues to increase, with the annual incidence in the US expected to double between 2011 and 2030 [51]. Recent survivors tend to be younger and have a favorable prognosis, so there will be a growing number of survivors in the future [17]. Survival is similar for patients with head and neck cancer regardless of the primary treatment modality (i.e., primary surgical vs. primary medical) [46, 47], so understanding the functional outcomes of therapy is important in guiding patient care.

Several studies have attempted to describe the functional impairment of patients who undergo TORS (particularly in comparison to primary radiation therapy and chemoradiotherapy); however, outcomes vary. The majority of these studies do not describe the physiology of dysphagia in this population and instead rely on patient‐reported symptoms and quality of life metrics [17]. These non‐physiologic metrics do not reliably correlate with physiologic impairment [28, 29, 30] and therefore cannot guide providers in developing targeted treatment for patients. Additionally, it is well‐known that swallowing impairments continue to evolve over time regardless of the treatment modality [3, 52, 53] To optimize treatment for patients in all phases of recovery, it is important to understand the evolution of dysphagia from the immediate post‐procedural period to months or years after the conclusion of therapy. In the present study, we sought to describe the physiologic impairments of head and neck cancer patients receiving TORS over time.

### 4.1 Perioperative Dysphagia After TORS

There is limited literature regarding dysphagia in the perioperative period after TORS [18]. One study, using EAT‐10, a validated subjective questionnaire for dysphagia severity, found that the majority of patients receiving TORS experience some dysphagia in the first post‐operative month (before adjuvant therapy), although 98% were able to start an oral diet by post‐op day 30 [18]. Another group sought to evaluate how TORS alone impacts swallowing using patient‐rated swallowing quality of life metrics as well as the Dynamic Imaging Grade of Swallowing Toxicity (DIGEST) scale, a validated score of swallow safety (airway protection) and efficiency (pharyngeal residue) using modified barium studies [19]. They found that patients undergoing TORS were functionally intact with swallow function near baseline at 1‐month post‐surgery based on normal safety and efficiency on DIGEST scores and high levels of patient‐reported eating and swallowing quality of life.

The present study contributes an evaluation of the physiologic impairment of patients in the perioperative period. At a mean of 27 days post‐op, we found a statistically significant proportion of patients experienced worsening of swallow function in component 10 (epiglottic movement) and component 11 (laryngeal vestibular closure) from baseline. The percentage of patients with a score of 0 in component 10, indicating complete epiglottic inversion (physiologically normal), dropped from 76% at baseline to 52% post‐operatively. For component 11, the percentage of patients who scored a 0 (physiologically normal) dropped from 66% to 31%, with 62% and 7% of patients showing mild and severe impairment, respectively (for further details, see the supporting information).

Consistent with our findings, Ottenstein et al. [40] also found the majority of patients to have impairments in epiglottic inversion and laryngeal vestibular closure 3 weeks after surgery. They postulated that impairments in laryngeal elevation may lead to impairments in epiglottic inversion [40]. They also found significant worsening in the immediate post‐operative period in components 12 (pharyngeal stripping wave), 13 (pharyngeal contraction), 15 (tongue base retraction), and 16 (pharyngeal residue) [40]. Another study in a heterogeneous population of patients with dysphagia referred to an ENT clinic evaluating the mechanics of epiglottic movement found that reduced tongue base contraction and laryngeal elevation can lead to impaired epiglottic inversion [54]. It is possible that the patients in our cohort experienced worsening in epiglottic inversion due to impaired tongue base retraction, which our small study did not have the power to detect.

Our data set was rather heterogeneous and time points for perioperative swallow studies ranged widely (with a standard deviation of 18 days). It is possible that studies performed very early after surgery are more likely to reflect post‐surgical inflammation and pain and do not reflect true impairment at the ~1 month mark. Further stratification of patients in the early vs. later perioperative period may be important to guide treatment needs.

The dataset in this study was small and heterogeneous, and the results should be evaluated with this in mind. However, based on our data and that of Ottenstein, many patients certainly can experience physiological impairment in swallow function after TORS. Further research should be performed to further elucidate impairment of the pharyngeal swallow after TORS in a larger subset of patients. Clinicians should have a high index of suspicion for dysphagia in a patient in the post‐operative period.

### 4.2 Long‐Term Dysphagia After TORS

Despite the push for avoidance or de‐intensification of adjuvant therapy, at present, at least half of TORS patients still require radiation therapy or chemoradiotherapy [47]. Used as primary treatment modalities for head and neck cancers, both radiation therapy and chemoradiotherapy have known adverse effects on swallowing [55, 56], likely due to a combination of fibrosis and neuropathy [57]. After TORS *and* adjuvant therapy, swallow function (measured both by patient‐reported quality of life metrics and clinical indicators such as the need for gastrostomy tube placement) has been shown to at least be temporarily worsened after adjuvant therapy at around the 3‐month post‐operative time point before approximating baseline function by 1 year after surgery [52, 53, 58]. Even so, adjuvant therapy is a predictor of poor swallowing function even a year after TORS [17], with one published study suggesting a dose‐dependent effect of adjuvant therapy [25].

Compared to baseline, we found a statistically significant proportion of patients worsened in component 11 (laryngeal vestibular closure) and the PAS score at our post‐adjuvant therapy time point. Notably, the percentage of patients with a PAS score of 1 or 2 (indicating either lack of penetration/aspiration OR mild penetration with subsequent ejection of penetrated material) dropped from 55% pre‐surgery to only 26% after surgery and adjuvant therapy. This is consistent with some of the existing literature which shows persistent dysphagia even a year out from surgery [24, 25]. Some studies have found patient‐reported outcomes to return to near baseline after a year [52, 53, 59], though it is possible that patients in these studies were experiencing physiologic impairment even if they did not subjectively experience it.

Interestingly, Barbon et al. found that patients treated with primary radiation therapy were more likely to have impairment in laryngeal vestibular closure at 3–6 months compared to those treated primarily with TORS [41]. It is possible that our findings of worsened laryngeal vestibular closure may have been more related to the evolving effects of radiation therapy rather than late effects of TORS. Future research can compare dysphagia among patients undergoing TORS with or without adjuvant therapy.

Compared to the perioperative period, we did not find any significant changes in the swallowing function further out from surgery (mean 219 days post‐op). Most likely, the limited power of the current study may have prevented us from identifying significant disturbances in the swallowing function after adjuvant therapy, and this should be further explored with analyses exploring larger groups of patients. It is thus possible, though unlikely, that patients never experienced any physiologic changes after adjuvant therapy. However, post‐adjuvant therapy MBS studies were completed an average of 219 days after surgery, so it is also possible that there was a worsening in the swallow function after adjuvant therapy, which was already improving, as has been previously reported [52, 53]. A third possible explanation could be that radiation‐induced physiologic changes were continuing to evolve in some of our patients and that physiologic impairment would be seen at a later time point, especially given that dysphagia has been reported to present more than 2 years after the conclusion of primary radiation therapy due to late side effects [3]. Further understanding of swallow physiology at different time points throughout oncologic treatment can guide specific management of dysfunction.

The clinical significance of these physiologic impairments in our patients is uncertain. Impairments in laryngeal vestibular closure and PAS could suggest a failure in patients to protect their airway and thus an increased risk of aspiration events and their sequelae. The prevalence of aspiration pneumonia in patients post‐TORS has been reported to be as low as 1.7% ranging up to 15% [60, 61, 62]. Future research must be done to evaluate the clinical consequences of dysphagia and aspiration, including the risk of aspiration events.

### 4.3 Importance of Baseline Swallow Function

In addition to swallowing impairment as a result of treatment, there is a relatively high prevalence of baseline swallow dysfunction among TORS patients; one study evaluating baseline swallow function in TORS patients found that over one‐third of patients have some pre‐treatment swallow dysfunction [26]. Importantly, baseline swallowing function before TORS predicts swallowing function after surgery, at least in the short term [26]. Pre‐TORS MBSImP deficit in pharyngeal stripping wave, swallow initiation, and clearing pharyngeal residue have been associated with post‐TORS airway invasion on PAS scores [40].

Here, we also found a relatively high prevalence of penetration and aspiration among TORS patients; 62% (18/29) of patients had penetration (score 2–5) and 7% (2/29) had aspiration (score 6–8) on the PAS scale at baseline. Every patient in our study had at least two MBSImP components with scores > 1 at baseline as well, indicating some level of impairment in swallowing physiology, even if it does not lead to penetration/aspiration or patient‐perceived impairment. Among patients who showed no worsening post‐treatment, many were impaired at baseline and remained so after cancer‐directed treatment. Even among patients who technically had improvements in MBSImP scores, many remained impaired (meaning they did not improve to a score of 1). Therefore, even if patients describe no worsening or even improvement in the swallowing function after treatment, they may still be impaired. Obtaining pre‐treatment swallowing metrics may assist clinicians in identifying patients at a higher risk of or worsening dysphagia after treatment, especially as the prevalence of baseline dysphagia in TORS patients is so high.

## 5 Limitations

There were several limitations to this study. For one, the power of the study was limited by the relatively small number of subjects included. True differences in swallowing dysfunction may have been missed as a result. For example, it is expected that patients with an extension of the tumor into the soft palate might have impairment in component 7 (soft palate elevation) after surgery. It should be noted that *p*‐values were not adjusted for the number of components measured. Components where we found a statistically significant change in score may have had a statistically insignificant *p*‐value if adjusted for the number of components. However, given the dearth of data on these measures for this population, we feel that these results are still worth presenting with this as an acknowledged limitation. In addition, the majority of the subjects did not have swallow studies completed at all three time points. For example, some patients only had a pre‐surgery and post‐surgery study, missing post‐adjuvant therapy studies. Others may have been missing their post‐surgery study but still had a pre‐surgery and post‐adjuvant therapy study completed. As such, the three time point comparisons (pre‐surgery to post‐surgery, pre‐surgery to post‐adjuvant, and post‐surgery to post‐adjuvant) cannot be directly compared as they could be if all patients had all time points. Our study was also limited by the lack of patient‐reported outcomes. Although an understanding of physiology can help to guide targeted treatment, patient understanding and quality of life are also of utmost importance.

There was significant variability of time points of videofluoroscopy within patient groups. For example, although the post‐surgery swallow studies were completed an average of 27 days post‐operatively, the standard deviation was high (18 days) with studies ranging from 4 days post‐op to 98 days post‐operatively. In the post‐adjuvant period, the range of time points of swallow studies was similarly high, from 100 to 425 days post‐surgery and 24–326 days post‐adjuvant therapy. Swallowing impairments likely are continuing to evolve over this time frame as patients continue to heal after surgery.

## 6 Conclusions and Next Steps

Both shortly after surgery and after completion of adjuvant therapy, patients have physiologic swallow dysfunction compared to baseline, particularly in components affecting airway protection. Many TORS patients also have baseline physiologic swallow impairment that persists after treatment. Further research is required to investigate the long‐term morbidity of dysphagia among patients receiving TORS alone or with adjuvant therapy. An investigation of subgroups of patients by tumor volume or extension of disease can help to guide specific therapy. Given the high prevalence of swallow dysfunction and the ability of physiologic assessment to guide targeted treatment, videofluoroscopic evaluation should be considered for all TORS patients at baseline and at later points during their treatment.

Studies rarely evaluate the functional impairment of TORS alone as they include patients with and without adjuvant therapy [17, 25, 52]. Although at present, over 80% of patients undergoing TORS receive adjuvant therapy [42], there has been a recent push for the de‐escalation of therapy in selected patients to optimize oncologic control while minimizing therapy‐associated morbidity [63]. A primary proposed strategy for de‐escalation is decreasing adjuvant therapy dose after surgical resection [63]. As such, evaluation of physiologic impairment at different time points for different treatment modalities and adjuvant doses is important in the development of safe and effective de‐escalation protocols.

## Author Contributions

All authors contributed to the study conception and design. Material preparation, data collection, and analysis were performed by A.C.W. and A.E.C. The first draft of the manuscript was written by A.C.W. and all authors commented on subsequent versions of the manuscript. All authors read and approved the final manuscript.

## Ethics Statement

This study was approved by the Northwestern University Institutional Review Board (STU00202162).

## Conflicts of Interest

The authors declare no conflicts of interest.

## Supporting information


Data S1.

## Data Availability

All data supporting the findings of this study are available within the paper and Data S1.
