# Supplementary material for: Short‐ and Long‐Term Swallowing Outcomes in Head and Neck Cancer Patients Receiving TORS and Adjuvant Therapy
Source: Head Neck. 2024 Dec 23;47(5):1345–54. doi: 10.1002/hed.28033 (PMC12038223; doi:10.1002/hed.28033)
Supplement: Supplementary file 1 — Data S1. [file HED-47-1345-s001.docx]

**Short and Long-Term Swallowing Outcomes in Head and Neck Cancer Patients Receiving TORS and Adjuvant Therapy**

Supplemental Material:

| Pre-Surgery |  |  |  |  |  |  |
| --- | --- | --- | --- | --- | --- | --- |
|  | 0 | 1 | 2 | 3 | 4 | Total Number Subjects |
| Component 1 | 0 (0) | 20 (69.0) | 8 (27.6) | 1 (3.4) | 0 (0) | 29 |
| Component 2 | 14 (48.3) | 10 (34.5) | 5 (17.2) | 0 (0) | 0 (0) | 29 |
| Component 3 | 19 (67.9) | 1 (3.6) | 8 (28.6) | 0 (0) | 0 (0) | 28 |
| Component 4 | 13 (44.8) | 1 (3.4) | 7 (24.1) | 8 (27.6) | 0 (0) | 29 |
| Component 5 | 0 (0) | 4 (13.8) | 22 (75.9) | 3 (10.3) | 0 (0) | 29 |
| Component 6 | 6 (20.7) | 8 (27.6) | 3 (10.3) | 12 (41.4) | 0 (0) | 29 |
| Component 7 | 24 (82.8) | 3 (10.3) | 2 (6.9) | 0 (0) | 0 (0) | 29 |
| Component 8 | 7 (24.1) | 21 (72.4) | 1 (3.4) | 0 (0) | 0 (0) | 29 |
| Component 9 | 1 (3.4) | 28 (96.6) | 0 (0) | 0 (0) | 0 (0) | 29 |
| Component 10 | 22 (75.9) | 6 (20.7) | 1 (3.4) | 0 (0) | 0 (0) | 29 |
| Component 11 | 19 (65.5) | 9 (31.0) | 1 (3.4) | 0 (0) | 0 (0) | 29 |
| Component 12 | 12 (41.4) | 16 (55.2) | 1 (3.4) | 0 (0) | 0 (0) | 29 |
| Component 13 | 26 (92.9) | 2 (7.1) | 0 (0) | 0 (0) | 0 (0) | 28 |
| Component 14 | 4 (13.8) | 24 (82.8) | 1 (3.4) | 0 (0) | 0 (0) | 29 |
| Component 15 | 0 (0) | 8 (27.6) | 19 (65.5) | 2 (6.9) | 0 (0) | 29 |
| Component 16 | 0 (0) | 11 (37.9) | 17 (58.6) | 1 (3.4) | 0 (0) | 29 |
| Component 17 | 6 (21.4) | 13 (46.6) | 7 (25.0) | 0 (0) | 2 (7.1) | 28 |

Figure 9. Distribution of MBSImP scores at the pre-surgery timepoint

| Post Surg |  |  |  |  |  |  |
| --- | --- | --- | --- | --- | --- | --- |
|  | 0 | 1 | 2 | 3 | 4 | Total Number Subjects |
| Component 1 | 0 (0) | 18 (62.1) | 9 (31.0) | 0 (0) | 2 (6.9) | 29 |
| Component 2 | 11 (37.9) | 11 (37.9) | 5 (17.2) | 2 (6.9) | 0 (0) | 29 |
| Component 3 | 16 (72.7) | 2 (9.1) | 4 (18.2) | 0 (0) | 0 (0) | 22 |
| Component 4 | 13 (44.8) | 0 (0) | 7 (24.1) | 8 (27.6) | 1 (3.4) | 29 |
| Component 5 | 0 (0) | 3 (10.3) | 22 (75.9) | 4 (13.8) | 0 (0) | 29 |
| Component 6 | 6 (20.7) | 5 (17.2) | 7 (24.1) | 11 (37.9) | 0 (0) | 29 |
| Component 7 | 16 (55.1) | 7 (24.1) | 5 (17.2) | 1 (3.4) | 0 (0) | 29 |
| Component 8 | 4 (13.8) | 21 (72.4) | 3 (10.3) | 1 (3.4) | 0 (0) | 29 |
| Component 9 | 0 (0) | 28 (96.6) | 1 (3.4) | 0 (0) | 0 (0) | 29 |
| Component 10 | 15 (51.7) | 10 (34.5) | 4 (13.8) | 0 (0) | 0 (0) | 29 |
| Component 11 | 9 (31.0) | 18 (62.1) | 2 (6.9) | 0 (0) | 0 (0) | 29 |
| Component 12 | 9 (31.0) | 17 (58.6) | 3 (10.3) | 0 (0) | 0 (0) | 29 |
| Component 13 | 27 (96.4) | 0 (0) | 1 (3.6) | 0 (0) | 0 (0) | 28 |
| Component 14 | 7 (24.1) | 18 (62.1) | 3 (10.3) | 1 (3.4) | 0 (0) | 29 |
| Component 15 | 0 (0) | 5 (17.2) | 16 (55.2) | 6 (20.7) | 2 (6.9) | 29 |
| Component 16 | 0 (0) | 4 (13.8) | 19 (65.5) | 4 (13.8) | 2 (6.9) | 29 |
| Component 17 | 6 (22.2) | 12 (44.4) | 6 (22.2) | 0 (0) | 3 (11.1) | 27 |

Figure 10. Distribution of MBSImP scores at the post-surgery timepoint

| Post-Adjuvant |  |  |  |  |  |  |
| --- | --- | --- | --- | --- | --- | --- |
|  | 0 | 1 | 2 | 3 | 4 | Total Number Subjects |
| Component 1 | 0 (0) | 13 (56.5) | 5 (21.7) | 2 (8.7) | 3 (13.0) | 23 |
| Component 2 | 12 (52.2) | 7 (30.4) | 3 (13.0) | 1 (4.3) | 0 (0) | 23 |
| Component 3 | 10 (45.5) | 1 (4.5) | 10 (45.5) | 1 (4.5) | 0 (0) | 22 |
| Component 4 | 7 (30.4) | 0 (0) | 6 (26.1) | 9 (39.1) | 1 (4.3) | 23 |
| Component 5 | 0 (0) | 2 (8.7) | 17 (73.9) | 4 (17.4) | 0 (0) | 23 |
| Component 6 | 1 (4.3) | 6 (26.1) | 4 (17.4) | 12 (52.2) | 0 (0) | 23 |
| Component 7 | 15 (65.2) | 7 (30.4) | 1 (4.3) | 0 (0) | 0 (0) | 23 |
| Component 8 | 2 (8.7) | 17 (73.9) | 3 (13.0) | 1 (4.3) | 0 (0) | 23 |
| Component 9 | 0 (0) | 23 (100) | 0 (0) | 0 (0) | 0 (0) | 23 |
| Component 10 | 17 (73.9) | 3 (13.0) | 3 (13.0) | 0 (0) | 0 (0) | 23 |
| Component 11 | 8 (34.8) | 14 (60.9) | 1 (4.3) | 0 (0) | 0 (0) | 23 |
| Component 12 | 8 (34.8) | 13 (56.5) | 2 (8.7) | 0 (0) | 0 (0) | 23 |
| Component 13 | 22 (95.7) | 0 (0) | 0 (0) | 1 (4.3) | 0 (0) | 23 |
| Component 14 | 3 (13.0) | 18 (78.3) | 2 (8.7) | 0 (0) | 0 (0) | 23 |
| Component 15 | 0 (0) | 3 (13.0) | 16 (69.6) | 3 (13.0) | 1 (4.3) | 23 |
| Component 16 | 0 (0) | 6 (26.1) | 13 (56.5) | 4 (17.4) | 0 (0) | 23 |
| Component 17 | 4 (17.4) | 7 (30.4) | 7 (30.4) | 0 (0) | 5 (21.7) | 23 |

Figure 11. Distribution of MBSImP scores at the post-adjuvant timepoint

|  | 1 | 2 | 3 | 4 | 5 | 6 | 7 | 8 | # Subjects |
| --- | --- | --- | --- | --- | --- | --- | --- | --- | --- |
| Pre-Surg | 9 (31.0) | 7 (24.1) | 11 (37.9) | 0 (0) | 0 (0) | 0 (0) | 1 (3.4) | 1 (3.4) | 29 |
| Post-Surg | 3 (10.3) | 5 (17.2) | 16 (55.2) | 0 (0) | 0 (0) | 0 (0) | 2 (6.9) | 3 (10.3) | 29 |
| Post-adjuvant | 3 (13.0) | 3 (13.0) | 14 (60.9) | 0 (0) | 1 (4.3) | 0 (0) | 1 (4.3) | 1 (4.3) | 23 |

Figure 12. Distribution of PAS scores at all timepoints
